# Personal librarian programs in medical and academic health sciences libraries: a preliminary study

**DOI:** 10.5195/jmla.2022.1290

**Published:** 2022-01-01

**Authors:** Natasha Audrey Williams

**Affiliations:** 1 natasha.williams@ucf.edu, User Services Librarian, Harriet F. Ginsburg Health Sciences Library. University of Central Florida College of Medicine. Orlando. FL

**Keywords:** personal librarian programs, outreach

## Abstract

**Objective:**

This preliminary study examined how personal librarian programs are implemented within medical and academic health sciences libraries. Increasing awareness of these programs and how they are implemented could create a larger and more accessible knowledge base for establishing best practices that similar libraries can look to when creating their own programs.

**Methods:**

To characterize existing programs, a twenty-two-item survey was sent to MEDLIB-L, AAHSL-ALL, ARCL-HSIG, and PSS-Lists email listservs in October 2018 to reach a broad audience of medical and academic health sciences librarians. Survey responses were analyzed using Qualtrics and Excel.

**Results:**

Of the 2,882 potential email recipients, 49 survey sessions were recorded, and a total of 38 survey sessions were completed (1.3% response rate). Of the 38 completed responses, representatives of 12 libraries (31.5%) reported that a personal librarian program had been implemented at their institution. For implementation, eight libraries involved 1–5 librarians, and four involved 6–10. Librarians were assigned 50–100 (n=6), 101–150 (n=1), or 151 or more (n=1) students each. The identified programs served medical students (n=11), nursing students (n=7), health professions students (n=7), dental students (n=2), and students in other fields (n=4). Services provided and communication methods were also identified.

**Conclusions:**

The personal librarian programs identified by the survey were uniquely structured to best meet the needs of their users, though similarities in implementation existed across institutions. Medical and academic health sciences libraries can look to these libraries as practical examples when starting their own personal library programs.

## INTRODUCTION

From a customer service perspective, libraries are in a unique position to build lasting relationships with their users due to the intimate nature of providing just the right information at just the right time. Gutek finds that more traditional “encounter-based” transactions and services are typically fleeting and can involve multiple service providers and argues that “relationship-based” transactions build upon each other due to repeated contact with the same providers [1]. In their discussion on the benefits of providing personalized library instruction and services to distance learners, Lillard notes that personalized approaches to providing library services can allow for the development of deeper relationships between academic librarians and students, particularly when compared to encounter-based interactions that are typical of most transactions in a library setting [2]. Using Gutek's concepts as a guide to define what an encounter-based service might look like in a library setting, Lillard offers the example of students constantly needing to redescribe their research projects to the reference librarian on duty each time they physically visit the library for assistance [2]. It would be better for students to work with one librarian throughout “the entire life of a research project or a course” [2], which would allow for a working relationship to form between the student and that one librarian. When this discussion is applied to methods for library outreach, this idea of relationship-building can be reflected by the concept of providing services through a personal librarian program. This preliminary study examines how personal librarian programs are implemented within medical and academic health sciences libraries.

### Personal librarian programs as a concept

Moniz notes that, historically, personal librarian programs are the result of ever-evolving changes to the profession, including new emphasis on information literacy and methods for best providing those skills, increased presence of digital and electronic resources, and even the need to find ways to retain students in higher education [3]. He defines the intent of a personal librarian program: “to build long-term, one-on-one connections that allow students to have the confidence and resources to be successful in the skill sets that librarians particularly seek to instill in them” [3]. While execution of a personal librarian program may vary from institution to institution, the basic setup remains the same. Nann explains: “Each incoming student is assigned a librarian. The librarian contacts the students at the beginning of his or her time at the school and at regular intervals” [4]. He goes on to explain that this concept differs from typical reference—like Lillard's encounter-based service—and other library programs due to this individualized approach and its potential ability to reach and build relationships with students who do not frequent the library [4]. In her chapter on development and implementation of personal librarian programs, Moats, like Nann, describes a standard execution: “In essence, personal librarians keep their students informed about resources and programs through periodic emails. They answer questions about library policies and procedures, assist with research strategies for projects, connect students with subject specialists, and support students when they are away from campus. Students are encouraged to meet in person with their assigned librarian. Personal librarians serve as a point of contact for students” [5].

Personal librarian programs are meant to individualize students' library experience. By assigning each student one librarian representative, students are connected with a go-to contact on whom they can rely for any library-related information they may need. Even if a student has never set foot in the library, the personal librarian acts as an ambassador for all library services and can seek to build a relationship simply by being the one familiar face and name the student associates with the library. While assigning one librarian as the primary contact for a set group of students suggests that the librarians in these programs could be overwhelmed with additional work, some librarians who have reported on their programs found that the overall rate of student participation in the program can be low and therefore an increase in workload is not experienced or may only be experienced during specific times throughout the academic year [6–8]. As Moats writes, “Depending on how receptive the students are to the program, the workload may increase slightly for the librarians” [5]. Thus, the utility of such programs and the relative busyness of the personal librarians tend to rely on if and how often students within the programs utilize them. Effective assessment of the success of such programs can be difficult to measure with low student participation, though there are various reports in the literature that students who take advantage of their programs report positive experiences related to receiving the assistance or information they needed and having built a personal connection with their assigned librarian [6–10]. One could say that success is relative to the goals of each program. Librarian buy-in may also be a barrier for institutions hoping to implement a program. For instance, while reference and instruction librarians may find the role to be a suitable extension of their typical duties due to the fact that they may have more regular interactions with students, other librarians who experience limited interactions with students or whose primary role in the library does not require them to provide reference assistance may have concerns that their skillsets do not align with participating in the program [7,11].

### Personal librarian programs in medicine

While the literature indicates that these programs are more prevalent in the undergraduate, transfer, and first-year graduate academic settings [12], there is also evidence of application of this concept in the medical or academic health sciences library setting. The oldest and most frequently cited example is the personal librarian program at the Cushing/Whitney Medical Library at Yale University. This program, which has been in existence since 1996, is often referenced by others as the model for other programs. As described by Spak and Glover in 2007, their mission was to encourage more personal contact between librarians and individual students, having noted the increased use of and comfort with computers and the web and a decline in traditional reference interactions and contact time. The program initially started out in 1996 with five librarians assigned to approximately twenty students each and by the 2005–2006 academic year adjusted to seven librarians assigned to ten to twenty students per class year. The program also expanded beyond medical students and serves other departments and educational programs at the medical center. The library's education services librarian acts as administrator for the program, obtaining class rosters and maintaining a collection of potential messages and timelines for delivery that their personal librarians can reference. Personal librarians write emails to their students to remind them of the services they can provide and respond to student requests for assistance with research or other questions they may have about library resources or services. The program performed an evaluation of its services and the experiences of students in 2006. Of the estimated 500 students who received an invitation to complete the survey, 146 completed the assessment. Ninety-five percent (n=139) of respondents confirmed they knew who their personal librarian was. These positive responses, along with other encouragingly positive responses to questions measuring student use of services, satisfaction with communication, and satisfaction with how student requests were responded to by their personal librarian, led the library to conclude that “the program is a useful and integral service that the library provides to medical center students” [6]. The library also found ways to make improvements to the program based on feedback from the survey, such as adding librarian photos to the program's website and introducing more formal opportunities to bring students and their personal librarians together [6].

Outside of Yale, an article was published in 2018 describing the personal librarian program at the University of Central Florida College of Medicine [7]. As a born-digital library, the program was introduced in 2013 to ensure primarily first- and second-year medical students would have reason to come into the library space and engage with the library team. The program initially utilized six librarians each assigned twenty students and now involves five with approximately twenty-four students each. Much of the program's organization, communication of important information, and marketing is managed through the library's public services department, which includes two personal librarians in order to achieve consistency in program implementation, though all participating librarians perform the regular duties of meeting with their students one-on-one when requested, responding to student emails, and providing assistance as necessary. The program has since seen its personal librarian groups integrated into two pieces of the undergraduate medical student curriculum: a research module that spans both the first and second years and a practice of medicine module that introduces first-year students to patient experiences. No formal evaluations of the program have been performed by the library, though one question included in an end-of-year survey performed in 2016 by the college's assessment office found that 83% of the 110 first-year students surveyed (n=91) either agreed or strongly agreed that they found the program to be an adequate resource or service, as did 68% of the 119 second-year students surveyed (n=80) [7].

Lastly, a more recent article briefly mentioning a personal librarian program at the Oakland University William Beaumont School of Medicine Medical Library was published in 2020 and touches on how one librarian involved in the program continued to interact with her students while the campus was shut down due to the COVID-19 pandemic [13]. The literature is otherwise sparse in this area. As the concept is not new and the benefits of such programs have been well documented, it is more likely that medical librarians are doing the work of personal librarians and not writing about it or are referring to their work in this area using another term.

The rationale for conducting this study was that increasing awareness of personal librarian programs in medical and academic health sciences libraries and how they are implemented could create a larger and more accessible knowledge base for establishing best practices that similar libraries can look to, should they be interested in creating their own programs.

## METHODS

### Development of instruments

A twenty-three-item survey was initially developed by the researcher using Qualtrics to gather data regarding program implementation for the study. A total of six people reviewed and pretested the survey, including the researcher's colleagues and mentor. Following revisions, the final survey included twenty-two items (Appendix A). The survey contained both multiple-choice and open-ended questions addressing program basics (e.g., location of program, librarian-to-student ratio, services provided) and was broken into the following blocks: general information, program information, services, communication, and wrap-up information. Following the first two questions that established whether respondents could continue the survey, the next nineteen questions addressed how each personal librarian program was run. The first twelve of those questions addressed implementation basics, while the remaining seven covered services and the communication of those services. The final survey question was included as a courtesy to allow representatives of libraries to choose how they would like the data they provided to be shared by the researcher.

The researcher also developed fourteen additional questions addressing other aspects of program implementation (e.g., costs, benefits, measures of success) intended to be used during interviews with survey respondents who consented to schedule an optional follow-up interview with the researcher (Appendix B).

The survey, interview questions, and other study documentation was submitted to the University of Central Florida Institutional Review Board (IRB) and were determined to be exempt from review.

### Procedure and participants

In order to deploy the survey and reach a broad audience of medical and academic health sciences librarians, a message explaining the purpose of the study, informing consent, and containing a link to the questionnaire was sent to the following library email listservs in October 2018: MEDLIB-L (1,734 subscribers), AAHSL-ALL (350 subscribers), ARCL-HSIG (696 subscribers), and PSS-Lists (102 subscribers).

A second reminder email was sent to the same listservs one month later. The survey was available for thirty-seven days. Survey respondents were also asked to contact the researcher via email if they would like to participate in an optional follow-up interview. DOI: dx.doi.org/10.5195/jmla.2022.1290

### Data analysis

The responses for complete and incomplete survey sessions were analyzed in Qualtrics and Excel. Responses to the open-ended survey question regarding services (item Q17 in Appendix A) were organized into four categories by the researcher based on similar themes that appeared in the responses.

## RESULTS

Of the 2,882 possible respondents reached across listserv subscribers, 49 survey sessions were recorded, and a total of 38 survey sessions were completed (1.3% response rate). Of the 38 completed responses, representatives of 12 libraries (31.5%) reported that a personal librarian program had been implemented at their institution (Table 1). When asked at the end of the survey to indicate how they would prefer for their responses to be shared by the researcher, five institutions requested that the specifics of their programs not be shared in a way that tied their responses directly to their programs. However, their data are still presented throughout the results in places where anonymous group data are discussed.

**Table 1:** Medical and academic health sciences libraries with personal librarian programs

Institution	Length of time program has been in existence	Approximate number of students served by program	Number of librarians in program	Number of students per librarian	Types of student served
Yale University Harvey Cushing/John Hay Whitney Medical Library	22 years	1,400	10	More than 50 (did not provide specific number)	Undergraduate medical students, postdoctoral medical students, undergraduate nursing students, graduate nursing students, doctoral nursing students (PhD), graduate health professions students, other
Oakland University William Beaumont School of Medicine	Specific institutional data omitted by request				
University of Massachusetts Medical School	9–10 years	301–400	6	100	Undergraduate medical students, graduate medical students, doctoral medical students (PhD)
University of Miami Miller School of Medicine	Specific institutional data omitted by request				
University of Central Florida College of Medicine	5–6 years	401–500	5	88	Undergraduate medical students
Walsh University	5–6 years	401–500	5	100	Undergraduate medical students, undergraduate nursing students, undergraduate dental students, undergraduate health professions students, other
Mount Saint Mary College	Specific institutional data omitted by request				
Redcliffe and Caboolture Hospital Libraries	20 years	201–300	1	100	Graduate medical students, doctoral medical students (PhD), postdoctoral medical students, undergraduate nursing students, graduate nursing students, undergraduate health professions students, graduate health professions students, doctoral health professions students (PhD)
University of Toledo	Specific institutional data omitted by request				
Ohio Northern University	3–4 years	2,800	5	More than 50 (did not provide specific number)	Undergraduate nursing students, undergraduate health professions students, other
UC San Diego	9–10 years	100–200	1	135	Undergraduate medical students
University of Florida	Specific institutional data omitted by request				

### Implementation basics

All libraries only utilized professional librarians in their programs (i.e., master's degree in library science or a related degree). Regarding the staffing required to run each library's program, eight libraries involved 1–5 librarians in their implementation, while four involved 610 librarians. Most librarians were assigned 50–100 students each (n=6), while one library reported that each librarian was assigned 101–150 students, and another library reported assigning each librarian 151 or more students; four libraries did not provide a response to this question. All but one library reported serving medical students in their personal librarian program (n=11). Nursing (n=7), health professions (n=7), dental (n=2), and students from other fields (n=4) were also served by these programs. A breakdown of student types can be seen in Table 2. Most students were enrolled in their personal librarian programs from matriculation to graduation (n=10). One library reported that their program only enrolled first-year undergraduates, and another enrolled students throughout their “whole tertiary study—between 2 and 6 years.”

**Table 2:** Types of students served by personal librarian programs

Area of study	Type of student
Medicine	Undergraduate (n=9) Graduate (n=4) Doctoral (PhD) (n=4) Postdoctoral (n=3)
Nursing	Undergraduate (n=7) Graduate (n=3) Doctoral (PhD) (n=2) Postdoctoral (n=1)
Health professions	Undergraduate (n=6) Graduate (n=3) Doctoral (PhD) (n=2) Postdoctoral (n=1)
Dental	Undergraduate (n=2) Graduate (n=1) Doctoral (PhD) (n=1) Postdoctoral (n=1)
Other	All other undergraduate students (n=1) All first year undergraduate students (n=1) Engineering Honors students (n=1) Business Honors students (n=1) Psychology Honors students (n=1) Education Honors students (n=1) Communication Honors students (n=1) Languages Honors students (n=1) Pre-Law Honors students (n=1) Graduate Public Health students (n=1) Physician Assistant students (n=1)

### Services

Respondents were asked to describe the services provided by their programs. Responses varied in length from bullet points copied and pasted from their library websites to written explanations. Services provided by personal librarians were generally found to fall under four categories that were determined after reading through the open-ended responses to item Q17 (Appendix A): research assistance, citation assistance, general library services, and other. See Tables 3–6 for specific answers by category. Services were advertised to students via one-on-one in-person interactions (n=9), in group settings (n=11), via email (n=12), through online reference services like Ask a Librarian (n=4), via the library website (n=10), over the phone (n=1), through the use of flyers (n=7) or brochures (n=3), and through social media channels like Facebook (n=4), Twitter (n=4), and Instagram (n=1). Two libraries provided alternate means of advertising services to their students, through their learning management system (LMS) (n=1), as well as during orientation and through the library's newsletter (n=1). These service advertisements occurred monthly for most libraries (n=9), whereas two libraries advertised their services one to two times weekly. One library reported daily advertisements.

**Table 3:** Research assistance services

Number of libraries represented: n=12
Assist with research by helping students: - articulate a good research or clinical question - identify the best sources to use for a project - create a methodologically sound search strategy - cite sources and write papers using EndNote
-Recommend information resources best suited for Capstone and other research needs -Help you with PubMed and other database searches
-Research Assistance -Literature Searching
Assistance finding information for research projects: -Articulating good research and/or clinical queries -Identifying the best databases to search -Using citation managers (EndNote, RefWorks, and/or Mendeley) -Finding additional resources
-Research assistance -Literature searching -editing for research paper
All Personal Librarians reach out and offer research and citation assistance
research support
-Research assistance -Literature searching -MeSH -Question development -Search terms -Recommendation on texts -Critical appraisal training -Research management
We offer our personal librarian service to students enrolled in the Honors program, as they often have more advanced research needs. Mostly this involves research assistance, literature searching, and use of EndNote software.
Research assistance which includes topic selection, finding sources, evaluating sources.
research assistance for their ISP (independent study projects), coaching for doing a systematic review
-Research Assistance -Literature Search -Non-predatory Journal Recommendations

**Table 4:** Citation assistance services

Number of libraries represented: n=8
- cite sources and write papers using EndNote
Using citation managers (EndNote, RefWorks, and/or Mendeley)
-Citation management
All Personal Librarians reach out and offer research and citation assistance.
citation support
EndNote training and assistance
-use of EndNote software
help with citing

**Table 5:** General library services

Number of libraries represented: n=8
Keep students informed with: - periodic emails highlighting resources and upcoming events - notices of extended hours Answer student questions about: - how to access books, articles, and more from collections - how to access library resources when students are off campus
Provide one-on-one instruction in the efficient and effective use of information resource
Organizing space for student projects
New resources and/or services in the Library: -New acquisitions (databases, book collections, etc.) -Special Library hours during exams and holidays -New study areas, facilities, or services Answer questions about Library services, policies, and procedures and other Library-related information: -Access Services (course reserves, electronic resources, printing, study rooms, etc.) -Document Delivery (finding e-journals, ordering articles through Interlibrary Loans, etc.) -Remote Access instructions -Mobile access to resources/creating personal accounts in databases, etc.
Email newsletters introducing services, resources, and more.
Provide basic assistance with general library services
remote access troubleshooting
-Information Resource Access -Instruction

**Table 6:** Other services

Number of libraries represented: n=7
Provide mentoring and support for self-directed learning
Teach/Serve as faculty for options courses
Help with specific curriculum activities relating to locating evidence based medicine resources for patient care
individual consultations
-Training -Journal Club set up -Database management
-Assistance with PBL look up topics - assistance with doing professional posters for summer research program -facilitating resource sharing for 3rd year clerkships
-Rounding

### Communication

Regarding communication between personal librarians and their students, librarians reported being available to provide services one-on-one in person (n=12), in group settings (n=11), via email (n=12), through an online reference service (n=7), by phone (n=11), via a voice or video call service like Skype (n=4), through Facebook (n=1), and through Twitter (n=2). Three libraries used other ways to communicate with students, including through lunch-and-learn sessions and “reminders during integrated sessions” (n=1) as well as through their LMS (n=2).

As no survey respondents contacted the researcher with an interest in participating in a follow-up interview, interview questions remained unanswered.

## DISCUSSION

This study provided a glimpse into the inner workings of personal librarian programs in medical and academic health science libraries. The survey identified twelve libraries that utilized a personal librarian program to connect with their students, with Table 1 showing how most responding libraries implemented their programs. Previous reports indicate that standard execution of a personal librarian program requires that at least one librarian be assigned to a group of students [4,5]. Data from all reporting libraries in this study align with this model, though the specifics regarding execution vary from institution to institution. For instance, eight of the twelve libraries reported that between one and five librarians were involved in their personal library programs. Upon examination of their responses, the librarian-to-student ratio is understandably different across institutions. Additional investigation is necessary in order to determine how feasible the workload is for each library, though one could speculate that some programs that have been in existence for longer have made adjustments over the years that are working for them and speak to program feasibility. As an older program, the program at Yale previously documented their staffing changes in the literature and noted that “there may be one to three [students] who make contact” and concluding that “the return on investment of time and resources for this program has been immeasurable” [6] with regard to work experienced by the librarians involved. Likewise, as a younger program, the University of Central Florida has also written that “all librarians involved with the [personal librarian program] have found the time necessary to participate in the program to be manageable,” going on to say that “not every student chooses to seek help from their librarian, so the [personal librarians] are not inundated with requests” [7].

The survey also showed variation in the types of students served by the personal librarian programs identified. A scoping review of personal librarian programs in academic libraries found that programs tend to target undergraduates, transfer, and first-year graduate students more than other groups [12]. However, the detailed breakdown of student types in Table 2 is an indication that personal librarian programs can be highly adaptable and organized to meet the needs of the institution and the students served by the institution. Thus, there appear to be not many hard “rules” when creating a program, outside of staying true to the traditional concept of what a personal librarian program is by specifically targeting students. For the libraries identified here, it would be worth discussing further what specific factors—if any—went into deciding which types of students would be served by the programs.

Perhaps unsurprisingly, a key service for all libraries that responded was to provide assistance with research in some capacity. Some libraries lumped related services such as help with citation management with their research assistance services, while others appeared to consider it a separate service. A difference in what research assistance encompasses is expected, as each institution tailors their services to their librarians' skill sets and the needs of their students. As noted in the literature, instructing students in the research process is often a key goal of personal librarian programs [5,14]. While this service is likely not exclusively performed in the setting of a personal librarian program, medical and academic health sciences librarians could be using these programs as a means to share this knowledge if they are otherwise unable to reach their learners. The Association of American Medical Colleges notes the importance of new physicians entering residency being equipped with the skills to both form a clinical question and know how to retrieve evidence to advance patient care, so much so that this was identified as a core competency [15]. As discussed in the literature and identified in this study, the personal librarian program at the University of Central Florida has been fortunate to have had their program integrated into courses that teach research skills and where and how to retrieve evidence-based information for patient care [7], but not every librarian across the health professions landscape will find themselves deeply involved in the teaching of these skills inside the classroom. Barriers are well documented and can include lack of faculty or student interest [16], limited time for instruction within the curriculum [17], or even a shift to more asynchronous or distanced-learning environments [18]. Offering instruction in how to search, critically appraise, and cite the medical literature and on related topics, like reference management software use, through personal librarian programs is just one of many solutions developed to deliver this key curriculum outside a one-shot classroom session.

The relevance and potential additional usefulness of these programs, particularly recently when students might be isolated due to academic programs shifting to remote instruction in response to the COVID-19 pandemic, cannot be overstated. Personal librarian programs across the academic landscape often rely on periodic emails as a popular method to communicate with the students they serve [5,12], facilitating attempts to check in with students and offer assistance or provide a reminder that someone is thinking about them. Aside from email, libraries identified in this study also noted being available via phone, voice/video call, and services like Ask a Librarian, all of which are well suited for use during remote work. Many of the services mentioned by the libraries in this study can also be offered remotely and online, which allows the library to remain more or less visibly operational to students even if the doors are not physically open. Libraries with personal librarian programs have already worked to make this shift in their programs in effort to make sure their students feel welcome while the library space is largely inaccessible [8,19], including the health sciences library at the Oakland University William Beaumont School of Medicine [13].

For the purposes of this study, it was necessary to exclude similar yet different models of “personalized” outreach services provided by librarians, namely embedded librarianship, where a librarian may be integrated into a specific course (usually in a virtual sense) [3], and liaison librarianship, where a librarian may be assigned to a specific department or faculty member [3]. As personal librarian programs are generally always targeted toward interactions with students, the explanation of research that was provided in the call for participants for this study aimed to filter responses that would not be relevant to the traditional definition of personal librarianship by including specific definitions for both embedded and liaison librarianship and indicating that these were not being examined. That is not to say that the work of these other outreach methods does not also include a level of relationship-building that is reminiscent of personal librarianship; it could be that medical and academic health sciences librarians doing this work are simply using different terms for the same approach to providing personal or individualized assistance to their student patrons. The embedded librarianship programs at the Medical College of Georgia at Augusta University are a relevant example of where the work being done could be considered similar to a personal librarianship approach. Their embedded librarians worked directly with students to provide personalized feedback on the use of appropriate evidence-based information resources during small group learning sessions, and their assessment of this partnership suggests that librarian presence and participation in these sessions allowed the librarians a “deeper understanding of the information needs of students” [20], which seems key to any good relationship-building process.

Using the demographic data for each library willing to share, an academic health sciences or medical library without its own personal library program could identify a library similar to their own from this preliminary study and use it as a model. While it is difficult to make a true assessment of whether a completely similar execution would be feasible without having additional context related to successes and challenges, the program at Yale, which has been in existence long enough that the literature already notes that other programs have used it as a model [21–23] and that an assessment concluded that it was effective [6], serves as a good example of a program that works. Effective evaluation and assessment of these programs is problematic even in academic library settings “due to inconsistency with reporting, low response rates, and issues with tracking statistics” [12], so further discussion with representatives of the libraries themselves would be necessary to make informed conclusions regarding complete program reproducibility.

This study has a few notable limitations. Though the survey was sent across four email listservs, response rates for the survey were low. This could be due to subscriber overlap across the listservs (e.g., a librarian subscribing to multiple listservs or colleagues all subscribing to the same listserv and allowing one colleague to respond on the institution's behalf) or self-selection based on how the researcher defined the target audience for the study. It could also be that the person best able to answer the questions from the survey was not a subscriber to any of the listservs that were targeted for distribution. Additionally, there were plans to perform follow-up interviews with librarians who completed the survey and were willing to answer additional questions, but these questions did not have an opportunity to be asked. The request for follow-up interviews may have gotten lost within the explanation of research that was sent to recruit survey participants, though a point was also made to remind those who did complete the survey to reach out to the researcher via email to conduct an optional interview; this reminder was included on the thank-you page of the survey. Study protocol and consent documents were written such that the researcher was unable to contact the participants without having to revise the present study and seek IRB approval or exemption again, which did not align with the timeline for completing the research. The hope was that the answers from the interview questions could provide other libraries interested in creating their own programs with fuller context regarding program implementation. The decision to not include these questions as open-ended essay response questions in the survey itself was based on the desire to keep the survey as short as possible and the belief that actual one-on-one discussion could yield richer information. Future follow-up from this project will include recruiting representatives from these libraries for in-depth discussion of their respective programs. Lastly, allowing survey respondents to decide how their data could be shared by the researcher resulted in having what may have been useful data omitted from the presentation of the results (e.g., Table 1), thus making some of the data less meaningful. The researcher hopes to see more written about these libraries from their own perspectives in the future so that the omitted data can be reported on their own terms.

This preliminary study sought to answer the question of how personal librarian programs are implemented within medical and academic health sciences libraries. These libraries have indeed been doing the work of providing services to their student learners through the use of personal librarian programs, and no two programs were exactly alike in their execution. The focus of this research was not on determining whether or how each program identified success; future follow-up research is needed to address this aspect of each program. This preliminary research can be used by other medical and academic health sciences libraries to explore the creation of their own programs. With some exceptions, each library profile can serve as a model for what a personal librarian program can look like, and the researcher recommends that interested librarians reach out to any of these programs directly for advice or with program-specific questions that were not addressed in this article or within the raw data associated with this study.

## Data Availability

Data associated with this article are available in the University of Central Florida institutional repository, STARS (https://stars.library.ucf.edu/datasets/1).

## SUPPLEMENTAL FILES

**Appendix A.** Personal librarian programs in medical and academic HSLs

**Appendix B.** Questions for follow-up interviews
