# Supplementary material for: Personal librarian programs in medical and academic health sciences libraries: a preliminary study
Source: J Med Libr Assoc. 2022 Jan 1;110(1):87–96. doi: 10.5195/jmla.2022.1290 (PMC8830399; doi:10.5195/jmla.2022.1290)
Supplement: Supplementary file 1 — Appendix A. Personal librarian programs in medical and academic HSLs [file jmla-110-1-87-s01.docx]

Personal Librarian Programs in Medical and Academic HSLs

Start of Block: General Information

Q1 Does your library currently have a personal librarian program?

- Yes (1)
- No (2)

Skip To: End of Survey If Does your library currently have a personal librarian program? = No

Display This Question:

If Does your library currently have a personal librarian program? = Yes

Q2 Please provide the name of your academic institution. Provide the full name, no abbreviations (e.g. *University of Central Florida* instead of *UCF*).

________________________________________________________________

| Page Break |  |
| --- | --- |

End of Block: General Information

Start of Block: Program Organization

Q5 The next section will ask some questions about your library's personal librarian program and how it works.

Q6 How long has your personal librarian program been in existence?

- Less than 1 year (1)
- 1 - 2 years (2)
- 3 - 4 years (3)
- 5 - 6 years (4)
- 7 - 8 years (6)
- 9 - 10 years (7)
- More than 10 years (5)

Display This Question:

If How long has your personal librarian program been in existence? = More than 10 years

Q7 If your program has been in existence for 10 or more years, please share that length of time here. (e.g. 15 years)

________________________________________________________________

Q8 Approximately how many students does your personal librarian program serve?

- Less than 100 students (1)
- 100 - 200 students (2)
- 201 - 300 students (3)
- 301 - 400 students (4)
- 401 - 500 students (5)
- 501 - 600 students (6)
- More than 600 students (7)

Display This Question:

If Approximately how many students does your personal librarian program serve? = Less than 100 students

| 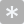 |
| --- |

Q18 If your program serves less than 100 students, please share that number here.

________________________________________________________________

Display This Question:

If Approximately how many students does your personal librarian program serve? = More than 600 students

| 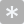 |
| --- |

Q9 If your program serves more than 600 students, please share that number here.

________________________________________________________________

Q10 What types of students does your program serve? Select all that apply.

- Undergraduate Medical students (1)
- Graduate Medical students (2)
- Doctoral Medical students (PhD) (3)
- Post-Doctoral Medical students (4)
- Undergraduate Nursing students (5)
- Graduate Nursing students (6)
- Doctoral Nursing students (PhD) (7)
- Post-Doctoral Nursing students (8)
- Undergraduate Dental students (9)
- Graduate Dental students (10)
- Doctoral Dental students (PhD) (11)
- Post-Doctoral Dental students (12)
- Undergraduate Health Professions students (13)
- Graduate Health Professions students (14)
- Doctoral Health Professions students (PhD) (15)
- Post-Doctoral Health Professions students (16)
- Other: (17) ________________________________________________

| Page Break |  |
| --- | --- |

Q11 Within your library, who participates in your personal librarian program as a personal librarian?

- Librarians (holds a masters in Library Science or related degree) (1)
- Library staff (does not hold a masters in Library Science or related degree) (2)
- Both librarians and library staff (3)

Display This Question:

If Within your library, who participates in your personal librarian program as a personal librarian? = Librarians (holds a masters in Library Science or related degree)

Or Within your library, who participates in your personal librarian program as a personal librarian? = Both librarians and library staff

| 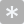 |
| --- |

Q12 How many librarians participate in your personal librarian program? Enter the number here.

________________________________________________________________

Display This Question:

If Within your library, who participates in your personal librarian program as a personal librarian? = Library staff (does not hold a masters in Library Science or related degree)

Or Within your library, who participates in your personal librarian program as a personal librarian? = Both librarians and library staff

| 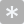 |
| --- |

Q13 How many library staff participate in your personal librarian program? Enter the number here.

________________________________________________________________

| Page Break |  |
| --- | --- |

Q15 Approximately how many students are assigned to each of the library participants in your program?

- Less than 10 students (1)
- 10 - 20 students (2)
- 21 - 30 students (3)
- 31 - 40 students (4)
- 41 - 50 students (5)
- More than 50 students (6)

Display This Question:

If Approximately how many students are assigned to each of the library participants in your program? = Less than 10 students

Or Approximately how many students are assigned to each of the library participants in your program? = More than 50 students

| 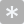 |
| --- |

Q30 How many students are assigned to each of the library participants in your program? Enter the number here.

________________________________________________________________

| Page Break |  |
| --- | --- |

Q31 How long are students enrolled in your personal librarian program?

- From matriculation to graduation (1)
- Another duration of time, please describe (e.g. *First two years of medical school*) (2) ________________________________________________

End of Block: Program Organization

Start of Block: Services

Q16 The next section will ask about the services your personal librarian program provides to the students it serves.

Q17 Please describe the services your personal librarian program offers to students. (e.g. Research assistance, Editing, Literature Searching, etc.)

________________________________________________________________

________________________________________________________________

________________________________________________________________

________________________________________________________________

________________________________________________________________

| Page Break |  |
| --- | --- |

End of Block: Services

Start of Block: Communication

Q20 This section will ask about how your program communicates with its students.

Q24 Does your personal librarian program tell students about the services it can provide?

- Yes (1)
- No (2)

Display This Question:

If Does your personal librarian program tell students about the services it can provide? = Yes

Q21 In what ways does your personal librarian program tell students about the services it can provide? Select all that apply.

- In person (one-on-one) (1)
- In person (group setting) (2)
- Email (3)
- Through an online reference service (e.g., Ask a Librarian) (4)
- Library website (5)
- Phone (6)
- Voice/Video call (e.g., Skype) (7)
- Facebook (8)
- Instagram (9)
- Twitter (10)
- Other social media (11) ________________________________________________
- Flyers (12)
- Brochures (13)
- Other (14) ________________________________________________

Display This Question:

If Does your personal librarian program tell students about the services it can provide? = Yes

Q23 How frequently does your personal librarian program actively tell students about the services it can provide?

- Daily (1)
- Weekly (2)
- Monthly (3)

Display This Question:

If How frequently does your personal librarian program actively tell students about the services it... = Daily

Q25 How many times daily?

- 1 -2 times a day (1)
- 3 - 4 times a day (2)
- 5 or more times a day (3)

Display This Question:

If How frequently does your personal librarian program actively tell students about the services it... = Weekly

Q26 How many times weekly?

- 1 - 2 times a week (1)
- 3 - 4 times a week (2)
- 5 or more times a week (3)

Q22 In what ways are your personal librarians available to communicate with students when providing services? Select all that apply.

- In person (one-on-one) (1)
- In person (group setting) (2)
- Email (3)
- Through an online reference service (e.g., Ask a Librarian) (4)
- Phone (5)
- Voice/Video call (e.g., Skype) (6)
- Facebook (7)
- Instagram (8)
- Twitter (9)
- Other social media (10) ________________________________________________
- Other (11) ________________________________________________

End of Block: Communication

Start of Block: Wrap-up information

Q28 As a reminder, by consenting to participate in this survey you have agreed to allow the researcher to document your responses so that they may be reported as data in publications, presentations, or other scholarly output. The next question will allow you to specify how you would like your responses to be shared by the researcher.

Q29 The researcher may share my responses in any publications, presentations, or other scholarly output regarding this study in the following ways. Select all that apply.

- My responses may be shared as part of a group of data not specific to my library (1)
- My responses may be shared as individual data not specific to my library (2)
- My responses may be shared as individual data specific to my library (3)

End of Block: Wrap-up information
