# Supplementary material for: Personal librarian programs in medical and academic health sciences libraries: a preliminary study
Source: J Med Libr Assoc. 2022 Jan 1;110(1):87–96. doi: 10.5195/jmla.2022.1290 (PMC8830399; doi:10.5195/jmla.2022.1290)
Supplement: Supplementary file 2 — Appendix B. Questions for follow-up interviews [file jmla-110-1-87-s02.docx]

**APPENDIX B**

**Questions for Follow-up Interviews**

| **Question Type** | **Specific Question and possible follow-up** |
| --- | --- |
| Creation of your program | 1. Where did your library learn about personal librarian programs? 2. Why did your library create their personal librarian program?    1. What needs were you trying to address? |
| Program set up | 1. How are students assigned to the librarians?    1. Is there a specific way the groups are assigned? 2. How does your library introduce students to the program? |
| Benefits to the library | 1. How has your library benefitted from having this program? 2. How have your personal librarians benefitted from their participation in this program? |
| Program Costs | 1. How would you describe the time commitment required to be a personal librarian in your program? 2. Are there any costs (aside from time spent) associated with running the program?    1. Does your program put on any events? |
| Assessment | 1. How does your program define “effectiveness” as it pertains to your personal librarian program? 2. Does your library measure whether or not your personal librarian program is effective?    1. In what ways are you measuring effectiveness? |
